# Natural Products and Neuroprotection 3.0

**DOI:** 10.3390/ijms24043885

**Published:** 2023-02-15

**Authors:** Cristina Angeloni, David Vauzour

**Affiliations:** 1 Department for Life Quality Studies, Alma Mater Studiorum, University of Bologna, Corso d’Augusto 237, 47921 Rimini, Italy; 2 Biomedical Research Centre, Norwich Medical School, University of East Anglia, Norwich NR4 7TJ, UK

In recent years, we have been witnessing a dramatic rise in the incidence of neurodegenerative diseases, a phenomenon partly associated with the increase in life expectancy. Alzheimer’s disease (AD), Parkinson’s disease (PD), amyotrophic lateral sclerosis (ALS), and multiple sclerosis (MS) are among the most common neurodegenerative disorders. They originate from the progressive deterioration of neuronal cells in different parts of the brain, and, because of the lack of regenerative ability of these cells, the damage inexorably leads to the loss of motor and cognitive functions. Although these pathologies have different clinical manifestations, they share common molecular mechanisms, such as abnormal protein aggregation, excitotoxicity, mitochondrial dysfunction, oxidative stress, and inflammation.

Despite decades of intense research, drug treatments for neurodegenerative diseases are limited, often offer only transient symptomatic relief, and are not considered disease-modifying. Given the social and economic burden caused by the rising frequency of these pathologies, there is an urgent need for the development of appropriate therapeutic strategies.

Due to the multifactorial etiology of these diseases, natural products, thanks to their pleiotropic biological activity, have attracted the interest of the scientific community. Natural compounds have been recognized to possess different bioactivities, including antioxidant, anti-inflammatory, and antiapoptotic effects. Moreover, natural compounds present the advantages of low toxicity and high tolerability.

This Special Issue reports on the impact of natural products on counteracting neurodegeneration and contains ten contributions addressing the most recent advances on this topic.

Several papers of this Special Issue focus on the potential neuroprotective effects of different polyphenols and their in vivo metabolites. 

Luteolin, a flavonoid majorly present in green pepper leaves and seeds, celery, and chamomile tea, has been suggested to possess neuroprotective activity in animal and cellular models of neurodegeneration. Ahmad et al. [1] focused on the potential protective effect of luteolin against amyloid-beta (Aβ1–42)-induced neuroinflammation, amyloidogenesis, and synaptic dysfunction in a mouse model of AD. Luteolin was able to inhibit the activation of c-Jun N-terminal kinases (p-JNK), p38 mitogen-activated protein kinases, glial fibrillary acidic protein (GFAP), and ionized calcium adaptor molecule 1 (IBA1) in the cortex and hippocampus of mice. Luteolin also attenuated inflammation reducing the expression of different inflammatory markers, reduced the expression of BACE-1 and Aβ1–42, and enhanced synaptic markers such as PSD-95 and SNAP-25. The authors suggested that the observed reduction of Aβ-associated neuroinflammation and neurodegeneration in luteolin-treated mice may be mediated by the inhibition of JNK. 

Curcumin, a polyphenol characterized by a yellow color, is found in the spice turmeric and has been widely investigated for its anti-inflammatory, antioxidant, and neuroprotective activities. In particular, studies have shown that curcumin is a positive allosteric modulator of α7-nicotinic acetylcholine receptors (nAChRs). As these receptors can modulate cognitive behavior, learning, and memory formation, α7-nAChRs could have a crucial role in neuro-pathological conditions such as autistic spectrum disorder (ASD) and attention-deficit hyperactivity disorder (ADHD). On these bases, Jayaprakash et al. [2] investigated the effects of curcumin in a rodent model of ASD. Curcumin potentiated the enhancement of spontaneous inhibitory postsynaptic currents induced by choline and ameliorated social deficits without affecting locomotor activity or anxiety-like behaviors. Moreover, curcumin increased the levels of superoxide dismutase and catalase in the hippocampus and the cerebellum reducing oxidative stress. Collectively, the observed results suggest that curcumin may represent a promising novel pharmacological strategy for ASD treatment.

El-Far et al. [3] further investigated the effects of curcumin and thymoquinone either alone or in combination in a rat model of ageing. Thymoquinone is a natural phytochemical isolated from *Nigella sativa* seeds with diverse biological activities, including antioxidant, anti-inflammatory, antidiabetic, and anticancer activities. Thymoquinone and curcumin significantly reduced oxidative alterations in the brain and heart tissues of treated rats, while their combination decreased elevated necrosis. Moreover, the thymoquinone and curcumin combination reduced the expression of genes associated with apoptosis and ageing. These results suggest that the combination of these two phytochemicals could be a promising approach in preventing ageing-related disorders.

Quercetin is a flavonoid present in fruits and vegetables such as apples, berries, cherries, red grapes, onions, and broccoli. Quercetin, like many other flavonoids, has several health-promoting effects, including anti-inflammatory, anti-mutagenic, anti-ischemic, anti-viral, anti-ageing, and antioxidant activity. As the main side effect of iron oxide nanoparticle (IONP) therapy is oxidative stress, especially at the brain level, Dora et al. [4]. investigated the antioxidant effect of quercetin supplementation in the brains of rats treated with IONPs for 30 days. Quercetin, in a dose-dependent manner, was able to counteract oxidative stress induced by IONPs through the reduction of malondialdehyde and the increase of glutathione and oxidized glutathione. In particular, quercetin was able to reverse severe brain tissue injuries due to iron deposition. Furthermore, quercetin induced a significant increase in brain epinephrine, serotonin, and melatonin through the upregulation of peroxisome proliferator-activated receptor gamma coactivator 1-alpha (PGC-1α) and mitochondrial transcription factor A (mtTFA). Quercetin was also able to counteract apoptosis in the brain, suggesting its supplementation is highly recommended in order to obtain the benefits of IONPs with fewer side effects.

Urolithin A is produced by the gut bacteria from ellagitannins, dietary polyphenols found in pomegranates and walnuts. Urolithin A has been shown to cross the blood–brain barrier and to evoke different biological activities. Esselun et al. [5] investigated the protective effect of urolithin A against mitochondrial dysfunction using SH-SY5Y-APP695 cells as a cellular model of early AD. Urolithin A did not modulate autophagy in SH-SY5Y-APP695 cells and had limited effects on mitochondrial function. Of note, the results suggested that Urolithin A triggers hormetic effects as it induces the transcription of several genes related to mitochondrial biogenesis.

Berberine, an isoquinoline alkaloid present in several plants, such as Berberidaceae, Papaveraceae, Menispermaceae, and Ranunculaceae, has been reported to have antibacterial, bile-production-enhancing, anti-inflammatory, and neuroprotective activities. With the aim of deepening the knowledge on the mechanisms underlying berberine neuroprotection, Szalak et al. [6] investigated the effect of the chronic administration of this compound on different stages of memory-related responses in mice. Berberine improved long-term memory acquisition in mice, an effect more likely related to an increase of parvalbumin-immunoreactive neurons (PV-IR) and nerve fibers in the hippocampal CA1-CA3 regions when compared to the control. Interestingly, berberine was detected in the brain and plasma, confirming its bioavailability. The authors suggested that berberine may modulate the level of Ca^2+^ in neurons and thus potentially act as a neuroprotective factor against neuronal damage.

Serra et al. [7], using a rat model of acute transient bilateral common carotid artery occlusion followed by reperfusion (BCCAO/R), investigated the effects of beta-caryophyllene in counteracting early hypoperfusion/reperfusion-induced damages. Beta-caryophyllene is a bicyclic sesquiterpene commonly found in the essential oils of many food plants and represents a primary component of the *Cannabis sativa* L. plant Rats were given a single dose of beta-caryophyllene before the BCCAO/R, and the levels of TRPV1, BDNF, trkB receptor, GFAP, and IBA1 were evaluated. The results of this study showed that the beta-caryophyllene pretreatment had modulatory effects on the ionotropic cannabinoid receptor TRPV1, the BDNF/trkB system, and the glial marker GFAP, suggesting that it could be an excellent therapeutic agent to counteract the pathophysiological sequelae of hypoperfusion/reperfusion damages.

*Hericium erinaceus* is an edible and medicinal mushroom that synthesizes many bioactive metabolites which are known to exert several health-promoting activities. Roda et al. [8], focusing on frailty, the geriatric syndrome associated with chronic systemic inflammation, investigated the effect of a standardized extract of *H. erinaceus* in aged mice. *H. erinaceus* partially recovered the age-related decline of locomotor performances, ameliorated cerebellar alterations, and reduced inflammation and oxidative stress while increasing SIRT1 and VEGF. These results suggest the efficacy of a non-pharmacological approach, such as the supplementation of *H. erinaceus* extract, as a promising adjuvant therapy to be associated with conventional geriatric treatments.

Young animal plasma has been considered as a potential therapeutic strategy for the treatment of neurodegenerative diseases thanks to encouraging results obtained in murine models for Alzheimer’s disease. Ruiz-Perera et al. [9] tried to translate these results from the murine to the human system, using inferior turbinate stem cells (ITSCs) as a model of age-associated neuronal degeneration in the adult human organism. They observed a strong neuroprotective activity of human plasma and human serum albumin against oxidative-stress-induced neuronal death on ITSCs. This neuroprotection was not observed using fetal bovine serum, suggesting that neuroprotection of human serum might be a species-dependent effect only observable with human serum. Moreover, they evidenced neuroprotection of plasma and human serum albumin against kainic-acid-induced excitatory stress in ex vivo cultured mouse hippocampal tissue slices. These results increase existing knowledge on the mechanisms underlying plasma-mediated neuroprotection, suggesting that further clinical studies should be carried out to develop new therapies based on the use of human plasma against neurodegenerative diseases

Leclerc et al. [10] wrote a very stimulating review with the aim of understanding whether natural products could directly act in the central nervous system, or whether their effect is due to an indirect effect associated with other mechanisms in the periphery. The authors focused on omega-3 polyunsaturated fatty acids from marine sources and polyphenols from plants. 

In conclusion, this Special Issue sheds new light on the potential protective effect of natural compounds in counteracting neurodegeneration, addressing natural compounds deriving from three different kingdoms: animals, plants, and fungi.
